# Health-related quality of life and its determinants among cancer patients: evidence from 12,148 patients of Indian database

**DOI:** 10.1186/s12955-024-02227-0

**Published:** 2024-03-13

**Authors:** Jyoti Dixit, Nidhi Gupta, Amal Kataki, Partha Roy, Nikita Mehra, Lalit Kumar, Ashish Singh, Pankaj Malhotra, Dharna Gupta, Aarti Goyal, Kavitha Rajsekar, Manjunath Nookala Krishnamurthy, Sudeep Gupta, Shankar Prinja

**Affiliations:** 1 Department of Community Medicine and School of Public Health, Post Graduate Institute of Medical Education and Research (PGIMER), Chandigarh, India; 2 Department of Radiation Oncology, Government Medical College and Hospital, Chandigarh, India; 3 Dr. B. Booroah Cancer Institute, Guwahati, Assam India; 4 Department of Medical Oncology, Adyar Cancer Institute, Chennai, Tamil Nadu India; 5 Department of Medical Oncology, All India Institute of Medical Sciences (AIIMS), New Delhi, India; 6 Department of Medical Oncology, Christian Medical College, Vellore, Tamil Nadu India; 7 Department of Clinical Haematology and Medical Oncology, Post Graduate Institute of Medical Education and Research (PGIMER), Chandigarh, India; 8 Department of Health Research, Ministry of Health and Family Welfare, New Delhi, India; 9 Department of Medical Oncology, Tata Memorial Centre, Mumbai, Maharashtra India

**Keywords:** Cancer, Health-related quality of life, Utility scores, Cancer site, Type of treatment, Treatment response, Adverse effects, India

## Abstract

**Background:**

Cancer survivors experience a decrement in health-related quality of life (HRQoL) resulting from the disease as well as adverse effects of therapy. We evaluated the HRQoL of cancer patients, stratified by primary cancer site, stage, treatment response and associated adverse events, along with its determinants.

**Methods:**

Data were collected from 12,148 patients, sampled from seven purposively chosen leading cancer hospitals in India, to elicit HRQoL using the EuroQol questionnaire comprising of 5-dimensions and 5-levels (EQ-5D-5L). Multiple linear regression was used to determine the association between HRQoL and various socio-demographic as well as clinical characteristics.

**Results:**

Majority outpatients (78.4%) and inpatients (81.2%) had solid cancers. The disease was found to be more prevalent among outpatients (37.5%) and inpatients (40.5%) aged 45–60 years and females (49.3–58.3%). Most patients were found to be in stage III (40–40.6%) or stage IV (29.4–37.3%) at the time of recruitment. The mean EQ-5D-5 L utility score was significantly higher among outpatients [0.630 (95% CI: 0.623, 0.637)] as compared to inpatients [0.553 (95% CI: 0.539, 0.567)]. The HRQoL decreased with advancing cancer stage among both inpatients and outpatients, respectively [stage IV: (0.516 & 0.557); stage III (0.609 & 0.689); stage II (0.677 & 0.713); stage I (0.638 & 0.748), *p* value < 0.001]. The outpatients on hormone therapy (B = 0.076) showed significantly better HRQoL in comparison to patients on chemotherapy. However, palliative care (B=-0.137) and surgery (B=-0.110) were found to be associated with significantly with poorer HRQoL paralleled to chemotherapy. The utility scores among outpatients ranged from 0.305 (bone cancer) to 0.782 (Leukemia). Among hospitalized cases, the utility score was lowest for multiple myeloma (0.255) and highest for testicular cancer (0.771).

**Conclusion:**

Older age, lower educational status, chemotherapy, palliative care and surgery, advanced cancer stage and progressive disease were associated with poor HRQoL. Our study findings will be useful in optimising patient care, formulating individualized treatment plan, improving compliance and follow-up.

## Introduction

Cancer has been reported to be one of the leading causes of mortality and disability in several countries [1, 2]. Recently, 19.3 million annual incident cases, and 10 million cancer deaths were reported globally [3]. In India, cancer caused 813,000 deaths, which was over 8% of total mortality [4]. Despite one-fourth of the incidence rate, mortality rates due to cancer in India as are high as in the developed countries [5]. This is attributable to several demand and supply side barriers to accessing treatment, [6–11] as a result of which more than half of the cancer patients are diagnosed in an advanced stage during their initial consultation with physicians [11–13]. This often leads to a poor quality of life for patients with advanced cancer, need for multimodality treatment, treatment related adverse effects and poor prognosis [14].

With increasing advancements in cancer treatment modalities such as chemotherapy, targeted therapy, radiotherapy, surgery etc., overall and progression-free survival among cancer patients have significantly improved. Quality of life in these cancer patients with prolonged survival becomes even more paramount. However, these therapeutic interventions usually precipitate with severe adverse effects that impact the overall quality of life in physical, psychological, and social dimensions. Previous reports have identified that cancer patients have a poorer health-related quality of life (HRQoL), which is attributable to both the disease and adverse effects (AEs) associated with treatment [15, 16]. Additionally, HRQoL tends to diminish with progression of disease and subsequent treatments [16, 17]. Therefore, beyond focusing on clinical efficacy and safety endpoints, it is crucial to incorporate HRQoL into treatment decision-making. This inclusion helps provide a more comprehensive understanding of the potential value of new therapies [18–24].

There are several detailed disease-specific instruments to measure HRQoL among cancer patients [25–27]. In contrast, generic instruments to assess HRQoL such as the EuroQoL five-dimensions five-levels (EQ-5D-5L) is an easy to administer tool to generate utility scores in the range of 0 (death) to 1 (full health) [28]. Such utility scores are suitable for estimating quality-adjusted life years (QALYs) in health technology assessments [29]. Quantitative assessments of HRQoL offer a means to assess the health outcomes from the patient’s viewpoint. Such estimates are valuable for health service planning and the formulation of health policies [30].

Overall, there is scarce evidence on HRQoL for cancer. Previous research has primarily focused on assessing HRQoL post-treatment or comparing pre- and post-treatment HRQoL [31–34]. While these assessments are valuable for understanding the treatment impact on HRQoL, establishing baseline data related to HRQoL in cancer patients based on factors such as primary site, cancer stage, type of treatment and treatment response is crucial for effective treatment planning, service provision, and follow-up of these patients [35]. The previous studies have either focused on single cancer type, or have certain methodological limitations [36–38]. None of the studies have reported cancer-site specific utility scores so far. Considering the gap in literature, the present study has comprehensively ascertained the HRQoL (stratified by primary cancer site, stage, treatment, treatment response, associated adverse events) among the nationally representative sample of 12,148 patients enrolled across 7 cancer hospitals in six states of India, using the generic EQ-5D-5L instrument [39]. Additionally, the factors influencing HRQoL were analysed using a regression analyses.

## Materials and methods

**Fig. 1:**
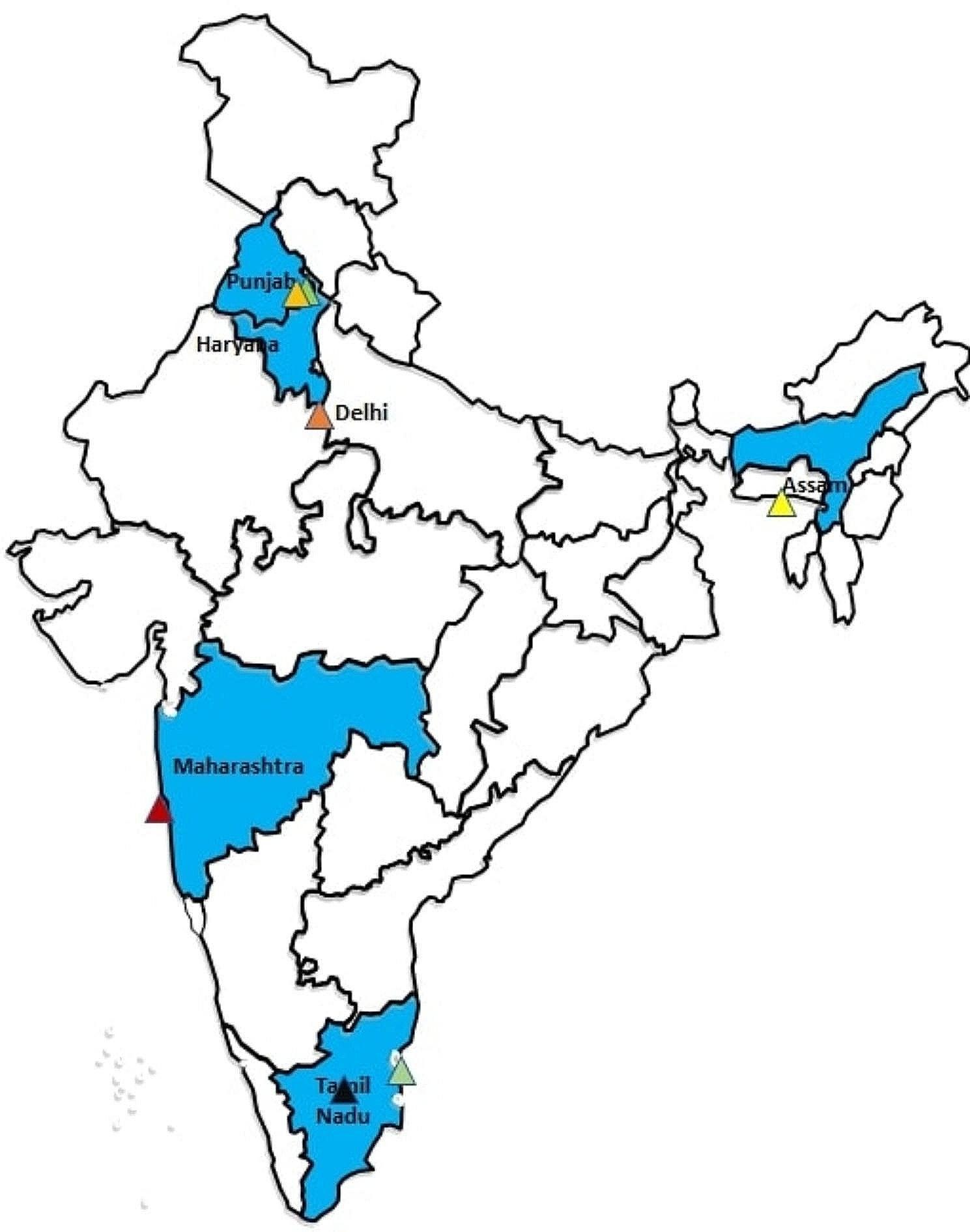
Selected study regions

The cancer patients were recruited from seven leading cancer hospitals (Fig. 1). It is worthwhile to mention that two-thirds of the chosen hospitals are among the top 10 hospitals in India, having highest number of cancer claims under India’s national health insurance scheme, Ayushman Bharat Pradhan Mantri Jan Arogya Yojana (ABPM-JAY) [40]. The detailed methodology has been documented in the protocol paper [39].

### Patient selection

#### Sample size and sampling

Patients were enrolled in a prospective manner during the period from October 2020 to March 2022 at the outpatient department (OPD) and inpatient department (IPD) of the chosen hospitals. Patients were recruited using a systematic random sampling technique, with the sampling interval determined by the average volume of patients per day in each hospital to obtain the required sample size. In the context of site-specific disease management groups (DMGs) clinics, probability proportional to size (PPS) method was used to determine the number of days when data was collected from respective DMGs [39]. On a particular day, when data was collected from a given DMG, a minimum of 10 patients were recruited. This method has ensured a minimum number of patients for each cancer site/type.

We aimed to interview a minimum of 1690 patients at each healthcare facility, resulting in a total sample of 11,830 across 7 health-care facilities. However, we were able to recruit 12,148 patients (9,787 in OPD and 2,359 in IPD) in India for HRQoL assessment.

#### Inclusion criteria

All cancer patients, irrespective of age and gender, who sought treatment at OPD and IPD at chosen hospitals were recruited. The OPD patients included: newly diagnosed individuals (recently diagnosed with cancer), on-treatment patients (currently undergoing active cancer treatments such as chemotherapy or radiotherapy), and follow-up cases (patients who have completed their treatment and are on follow-up, with or without maintenance therapy).

All the inpatient admissions who had stayed overnight in the hospital were recruited. The HRQoL was assessed on the day of recruitment. The case definitions used for enrolment of patients in OPD and IPD are outlined in Annexure I.

#### Assessment for quality of life

The EQ-5D-5L questionnaire includes five domains: mobility, self-care, usual activity, pain/discomfort, and anxiety/depression [28]. Each domain is graded on a scale of five levels, ranging from no problems to extreme problems. The Indian value set was used to derive the utility scores [42]. A score of ‘1’ indicates perfect health, and ‘0’ indicates death, with a range of 1 to -0.549 [43]. Additionally, patients were asked to rate their present health state on a scale from 0 to 100 through the EuroQol visual analogue scale (EQ-VAS) [44].

### Data analysis

We used Indian tariff value set to compute utility scores [45]. Mean utility scores according to primary cancer site, cancer stage, and type of treatment such as radiotherapy, chemotherapy, hormonal therapy, surgery, combination therapy etc. were calculated. We constructed the weights for different type of cancers using global disease burden data in the Indian context, in order to derive utility scores [46]. Sampling weights were calculated in order to correct for any discrepancies between the sample and the population. The sampling weights were computed for each stratum and were calculated as the ratio of the population count to the sample count within that stratum.

These analytical weights were then applied to give more or less weight to each observation according to their relative representativeness in the population. The weight for an individual in a particular stratum is equal to the total known population size in that stratum divided by the sample size in that stratum. The post-stratification weight, $$ {w}_{i}$$ for each respondent “i” in stratum “h” is calculated as:

$$ {w}_{i}=\raisebox{1ex}{${N}_{h} $}\!\left/ \!\raisebox{-1ex}{${n}_{h} $}\right.$$

where, $$ {N}_{h}$$ is known population size in stratum h and $$ {n}_{h}$$ is the sample size in stratum h.

Association between HRQoL and socio-demographic & clinical characteristics was assessed among cancer patients seeking outpatient as well as inpatient treatment.

### Assessment of factors associated with HRQoL

ANOVA test was applied to evaluate the statistical significance of difference in quality of life scores among patients of different age groups, religion, marital status, area of residence, educational status, income status (based on consumption expenditure) and clinical characteristics. An independent samples t-test was specifically used to assess differences in HRQoL with gender stratification. Using the data for OPD and IPD patients separately, we used the multivariable linear regression. The multiple linear model was assumed to be

1 $$ Y={b}_{0}+{b}_{1}{X}_{1}+{b}_{2}{X}_{2}+\dots +{b}_{k}{X}_{k}+e$$

where *Y* is the outcome variable, $$ {X}_{i}$$ is the value of the *i*^th^ predictor, and *e* is the error.

We used quality of life as a dependent variable, while remaining variables such as patient’s age, gender, area of residence, education level, marital status, wealth quintile, line of treatment, type of cancer and treatment, response to treatment, and occurrence of any adverse effect were considered as predictors. Normality of error term for both models (outpatient and inpatient) has been checked using “Kolmogorov Smirnov Test” with insignificant *p*-values as 0.253 (outpatients) and 0.185 (hospitalized cases). While the presence of homoscedasticity is checked using “Breusch-Pagan Test” with insignificance p-values as 0.098 (outpatients) and 0.188 (hospitalized cases), which fails to reject the null hypothesis of homoscedasticity. Hence, both models met the assumptions of a normal error term and homoscedasticity. There is no multicollinearity with variance inflation values ranging between 1.03 and 3.95 for outpatients, and from 1.06 to 2.03 for inpatients.

### Ethical considerations

Ethical approval was obtained individually from the Institute Ethics Committee of all the participating centres. A written informed consent was obtained from all study participants. In case of participants aged 18 years and above, consent was obtained directly from them. However, for participants below the age of 18, parental or guardian consent was sought.

## Results

The HRQoL of 9,787 cancer patients receiving outpatient care and 2,359 hospitalized cases was evaluated using the EQ-5D-5L and EQ-VAS tools. As shown in Table 1, Out of 9,787 outpatient cancer cases, majority were found to be diagnosed with solid cancers (78.4%) followed by haematological cancers (21.6%). At the time of recruitment, majority patients were in stage III (40.6%) and stage IV (29.4%) of cancer. Nearly 83.6% were on first line of treatment followed by 14.1% on second line treatment. The most common treatment modality at outpatient setting was chemotherapy and/or targeted therapy, given to approximately 50% cancer patients. The adverse effects were reported by 90% cancer patients.

**Table 1:** Mean utility scores among different socio-economic and clinical groups of cancer patients seeking outpatient treatment in India

Sociodemographic Category	Sample size	Mean HRQoL score		VAS Score	
	N (%)	Mean (95%CIs)	***p***-value*	Mean (95%CIs)	***p***-value*
***Age groups (in years)***					
0–15	312 (2.9%)	0.593 (0.548,0.639)	**< 0.001**	60.24 (58.22,62.26)	**< 0.001**
16–30	754 (7.1%)	0.728 (0.706,0.75)		65.66 (64.26,67.06)	
31–45	2566 (24.1%)	0.673 (0.659,0.686)		64.48 (63.77,65.19)	
45–60	4317 (40.6%)	0.632 (0.621,0.643)		63.66 (63.16,64.16)	
Above 60	2688 (25.3%)	0.563 (0.548,0.578)		62.39 (61.75,63.02)	
***Gender***					
Male	5050 (47.5%)	0.607 (0.597,0.617)	**< 0.001**	62.39 (61.92,62.86)	**< 0.001**
Female	5587 (52.5%)	0.651 (0.642,0.66)		64.65 (64.19,65.11)	
***Area of Residence***					
Urban	3464 (32.6%)	0.686 (0.674,0.697)	**< 0.001**	66.08 (65.44,66.71)	**< 0.001**
Rural	6997 (65.8%)	0.605 (0.597,0.614)		62.27 (61.88,62.65)	
Slum	175 (1.6%)	0.52 (0.456,0.584)		66.47 (63.99,68.95)	
***Education***					
No education	2455 (23.1%)	0.529 (0.513,0.545)	**< 0.001**	61.05 (60.39,61.7)	**< 0.001**
Primary & Middle	3806 (35.8%)	0.622 (0.611,0.633)		62.67 (62.14,63.2)	
Up to Senior Secondary	3117 (29.3%)	0.681 (0.67,0.693)		64.84 (64.22,65.46)	
Graduation & above	1259 (11.8%)	0.724 (0.706,0.742)		68.12 (67.07,69.18)	
***Wealth Quintile***					
Poorest	2112 (19.9%)	0.725 (0.712,0.737)	**< 0.001**	63.82 (63.04,64.6)	0.26
Poor	2027 (19.1%)	0.692 (0.677,0.706)		63.08 (62.29,63.88)	
Middle	2175 (20.4%)	0.606 (0.591,0.622)		64.09 (63.4,64.78)	
Rich	2249 (21.1%)	0.548 (0.532,0.564)		63.17 (62.5,63.83)	
Richest	2073 (19.5%)	0.587 (0.57,0.604)		63.71 (62.94,64.49)	
***Marital Status***					
Unmarried	904 (8.5%)	0.688 (0.665,0.71)	**< 0.001**	64.17 (62.92,65.42)	0.196
Married	8541 (80.3%)	0.633 (0.626,0.641)		63.62 (63.25,63.99)	
Separated/Divorced	53 (0.5%)	0.644 (0.562,0.727)		65.63 (61.59,69.66)	
Widow/Widower	1138 (10.7%)	0.559 (0.537,0.581)		62.71 (61.8,63.62)	
***Type of Cancer***					
Solid	9020 (86.7%)	0.629 (0.622,0.637)	**< 0.001**	63.25 (62.9,63.61)	**0.001**
Haematological	1387 (13.3%)	0.672 (0.654,0.691)		64.88 (63.88,65.89)	
***Type of treatment***					
Chemotherapy	3695 (41.8%)	0.644 (0.632,0.655)	**< 0.001**	60.5 (59.97,61.03)	**< 0.001**
Radiotherapy	601 (6.8%)	0.611 (0.582,0.64)		59.42 (58.18,60.65)	
Palliative Care	279 (3.2%)	0.54 (0.489,0.59)		52.64 (50.61,54.67)	
Surgery	649 (7.3%)	0.529 (0.501,0.557)		63.73 (62.6,64.86)	
Combination therapy**	1137 (12.9%)	0.604 (0.583,0.625)		62.83 (61.89,63.77)	
Maintenance therapy	143 (1.6%)	0.741 (0.703,0.779)		68.56 (65.09,72.04)	
Diagnostic	99 (1.1%)	0.686 (0.617,0.756)		56.53 (53.54,59.52)	
Hormone Therapy	231 (2.6%)	0.801 (0.769,0.833)		71.89 (69.8,73.99)	
Others	2002 (22.7%)	0.768 (0.755,0.782)		70.36 (69.5,71.22)	
**Cancer Stage**					
Carcinoma in Situ	4 (0.1%)	0.811 (0.514,1.107)	**< 0.001**	68.65 (40.27,97.02)	**< 0.01**
Stage I	463 (7.6%)	0.748 (0.724,0.772)		67.46 (65.85,69.07)	
Stage II	1221 (20.1%)	0.713 (0.697,0.728)		62.29 (61.34,63.25)	
Stage III	2311 (38%)	0.689 (0.676,0.701)		63.38 (62.7,64.06)	
Stage IV	2077 (34.2%)	0.557 (0.54,0.574)		59.05 (58.36,59.75)	
**Response to Treatment**					
Disease Free	2444 (28.2%)	0.691 (0.676,0.705)	**< 0.001**	71.35 (70.65,72.05)	**< 0.001**
Progressive Disease	442 (5.1%)	0.553 (0.513,0.593)		56.36 (54.53,58.2)	
Ongoing treatment*	5771 (66.7%)	0.638 (0.629,0.647)		60.76 (60.35,61.17)	
**Line to Treatment**					
First Line	7205 (84.9%)	0.653 (0.645,0.661)	0.012	63.75 (63.36,64.15)	**< 0.001**
Second Line	1133 (13.3%)	0.615 (0.592,0.638)		60.21 (59.18,61.25)	
Third Line	137 (1.6%)	0.634 (0.572,0.695)		63.42 (60.58,66.27)	
Fourth Line	14 (0.2%)	0.717 (0.514,0.921)		70 (60.23,79.78)	
Other	2 (0%)	0.873 (-1.588,3.334)		70.95 (-67.92,209.81)	
**Adverse Effect**					
With Adverse Effect	5300 (88.7%)	0.627 (0.618,0.636)	**< 0.001**	59.05 (58.63,59.47)	**< 0.001**
Without Adverse Effect	678 (11.3%)	0.835 (0.818,0.852)		73.28 (71.96,74.6)	
**Total**	**9787**	0.630 (0.623, 0.637)		63.58 (63.25, 63.91)	

*Level of significance at p- value less than 0.05, **Combination therapy– Chemotherapy + Radiotherapy, Surgery + Radiotherapy, Surgery + Chemotherapy, Surgery + Chemotherapy + Radiotherapy. Ongoing treatment-response cannot be assessed among patients on active oncology treatment

As shown in Table 2, among hospitalized cases (*N* = 2,359), maximum patients fall within the 45–60 years age group, accounting for 37.5%, followed by the 31–45 years category (23.3%) and those above 60 years (21%). Majority patients were found to be hospitalized in semi-private hospitals (69.1%) while 30.9% were admitted in public health care facilities. The duration of hospitalization in most of the patients (29.6%) was more than 5 days. Majority hospitalized cancer cases presented in stage III (40%) and IV (37.3%) at the time of recruitment. Detailed sample characteristics of outpatient and hospitalized cases are outlined in Tables 1 and 2 respectively.

**Table 2:** Mean utility scores among different socio-economic and clinical groups of indoor cancer patients in India

Sociodemographic Category	Sample size	Mean HRQoL score		VAS Score	
	N (%)	Mean (95%CIs)	***p***-value*	Mean (95%CIs)	***p***-value*
***Age groups (in years)***					
0–15	134 (5%)	0.606 (0.535,0.677)	**0.003**	55.03 (52.38,57.68)	0.342
16–30	254 (9.5%)	0.619 (0.573,0.666)		56.54 (54.57,58.51)	
31–45	620 (23.3%)	0.564 (0.537,0.591)		55.16 (54.04,56.29)	
45–60	1039 (39.1%)	0.54 (0.517,0.563)		56.59 (55.69,57.49)	
Above 60	613 (23%)	0.524 (0.495,0.554)		55.96 (54.77,57.16)	
***Gender***					
Male	1470 (55.2%)	0.551 (0.532,0.57)	0.805	56.44 (55.7,57.19)	0.112
Female	1191 (44.8%)	0.555 (0.533,0.576)		55.52 (54.65,56.39)	
***Area of Residence***					
Urban	1080 (40.6%)	0.572 (0.55,0.594)	0.092	55.75 (54.83,56.66)	**0.001**
Rural	1536 (57.7%)	0.54 (0.521,0.558)		56 (55.27,56.72)	
Slum	45 (1.7%)	0.541 (0.445,0.637)		63.99 (59.73,68.24)	
***Education***					
No education	471 (17.7%)	0.463 (0.427,0.5)	**< 0.001**	57.44 (56.14,58.73)	**< 0.01**
Primary & Middle	836 (31.4%)	0.556 (0.531,0.582)		55.03 (54.07,55.98)	
Up to Senior Secondary	802 (30.2%)	0.548 (0.523,0.573)		54.82 (53.76,55.88)	
Graduation & above	551 (20.7%)	0.63 (0.602,0.658)		58.1 (56.79,59.42)	
***Wealth Quintile***					
Poorest	506 (19%)	0.567 (0.536,0.598)	0.705	53.18 (51.92,54.44)	**< 0.001**
Poor	547 (20.6%)	0.558 (0.527,0.589)		54.87 (53.64,56.11)	
Middle	557 (20.9%)	0.556 (0.527,0.586)		56.37 (55.18,57.57)	
Rich	546 (20.5%)	0.547 (0.517,0.578)		58.21 (57.08,59.34)	
Richest	505 (19%)	0.535 (0.498,0.571)		57.4 (55.92,58.89)	
***Marital Status***					
Unmarried	318 (11.9%)	0.639 (0.598,0.679)	**< 0.001**	56.72 (54.98,58.46)	0.065
Married	2110 (79.3%)	0.547 (0.532,0.563)		55.68 (55.05,56.3)	
Separated/Divorced	22 (0.8%)	0.511 (0.286,0.736)		58.89 (49.78,68)	
Widow/Widower	211 (7.9%)	0.482 (0.426,0.538)		58.21 (56.15,60.28)	
***Type of hospital***					
Public	858 (32.3%)	0.379 (0.353,0.405)	**< 0.001**	62.36 (61.43,63.29)	**< 0.001**
Semi-Private	1802 (67.7%)	0.635 (0.62,0.651)		53.01 (52.35,53.68)	
***Duration of hospitalisation (days)***					
1	503 (18.9%)	0.663 (0.645,0.68)	**< 0.001**	50.49 (49.44,51.54)	**< 0.01**
2	291 (10.9%)	0.573 (0.528,0.618)		64.24 (62.33,66.14)	
3	290 (10.9%)	0.479 (0.434,0.524)		61.72 (60.2,63.24)	
4	348 (13.1%)	0.539 (0.498,0.58)		56.81 (55.35,58.28)	
5	435 (16.4%)	0.531 (0.495,0.567)		55.2 (53.85,56.55)	
> 5	794 (29.8%)	0.52 (0.491,0.549)		54.57 (53.5,55.64)	
***Type of cancer***					
Solid	2406 (90.4%)	0.56 (0.546,0.575)	**0.002**	56.36 (55.78,56.95)	**< 0.001**
Haematological	255 (9.6%)	0.483 (0.427,0.539)		52.88 (50.82,54.95)	
***Type of treatment***					
Chemotherapy	1646 (62.7%)	0.587 (0.57,0.603)	**< 0.001**	57.37 (56.69,58.05)	**< 0.001**
Radiotherapy	121 (4.6%)	0.621 (0.561,0.681)		54.65 (52.27,57.03)	
Palliative Care	39 (1.5%)	0.247 (0.122,0.372)		50.23 (44.72,55.74)	
Surgery	115 (4.4%)	0.518 (0.444,0.593)		57.99 (55.49,60.5)	
Combination therapy**	231 (8.8%)	0.413 (0.359,0.467)		58.92 (56.91,60.93)	
Maintenance therapy	16 (0.6%)	-0.276 (-0.425,-0.128)		27.11 (18.35,35.88)	
Diagnostic	110 (4.2%)	0.451 (0.369,0.534)		54.44 (51.21,57.68)	
Hormone Therapy	3 (0.1%)	0.488 (0.488,0.488)		60 (60,60)	
Immunotherapy	31 (1.2%)	0.726 (0.675,0.777)		51.31 (47.45,55.18)	
Others (clinical follow up)	311 (11.9%)	0.569 (0.527,0.611)		50.3 (48.56,52.04)	
**Cancer Stage**					
Carcinoma in Situ	1 (0%)	0.737 (0,0)	**< 0.001**	45 (0,0)	0.462
Stage I	83 (5%)	0.638 (0.573,0.704)		55.99 (52.36,59.62)	
Stage II	283 (17%)	0.677 (0.643,0.711)		55.82 (54.3,57.33)	
Stage III	599 (36%)	0.609 (0.583,0.635)		57.29 (56.02,58.56)	
Stage IV	698 (41.9%)	0.516 (0.489,0.543)		56.07 (55.05,57.08)	
**Response to Treatment**					
Disease Free	237 (9.1%)	0.553 (0.507,0.599)	**< 0.001**	53.36 (51.39,55.34)	**0.001**
Progressive Disease	131 (5%)	0.42 (0.345,0.496)		53.42 (50.42,56.43)	
Ongoing treatment*	2233 (85.9%)	0.561 (0.546,0.577)		56.63 (56.02,57.24)	
**Line to Treatment**					
First Line	2270 (87.4%)	0.572 (0.557,0.587)	**< 0.001**	56.33 (55.72,56.94)	**0.001**
Second Line	278 (10.7%)	0.417 (0.368,0.467)		55.51 (53.64,57.39)	
Third Line	36 (1.4%)	0.444 (0.308,0.579)		55.64 (50.45,60.83)	
Fourth Line	12 (0.5%)	0.416 (0.268,0.565)		39.27 (30.66,47.88)	
**Adverse Effect**					
With Adverse Effect	2097 (82.6%)	0.544 (0.528,0.56)	**< 0.001**	56.55 (55.93,57.17)	0.154
Without Adverse Effect	440 (17.4%)	0.637 (0.606,0.669)		55.35 (53.83,56.88)	
**Total**		0.553 (0.539,0.567)		56.03 (55.46,56.6)	

*Level of significance at p- value less than 0.05, **Combination therapy– Chemotherapy + Radiotherapy, Surgery + Radiotherapy, Surgery + Chemotherapy, Surgery + Chemotherapy + Radiotherapy. Ongoing treatment-response cannot be assessed among patients on active oncology treatment

### Cancer related HRQoL by socioeconomic factors

The mean utility score for outpatient and inpatient cancer patients across different socioeconomic and clinical groups are depicted in Tables 1 and 2 respectively. The mean utility score was significantly higher among cancer patients seeking outpatient care [0.630 (95% CI: 0.623, 0.637)] than those on inpatient treatment [0.553 (95% CI: 0.539, 0.567)]. Similarly, the mean EQ-VAS score [63.58 (95% CI: 63.25, 63.91)] was higher among outpatients than inpatients [56.03 (95% CI: 55.46, 56.6)].

Among outpatients, females had higher utility score (0.651) as compared to males (0.607). Similar trends were observed among hospitalized cancer patients, with an EQ-5D-5L index of 0.555 among females and 0.551 among males. Patients aged 16–30 years demonstrated the highest quality of life score, with a mean EQ-5D-5L score of 0.728 among outpatients and 0.619 among hospitalized cases. The utility scores among outpatients were found to be significantly lower among older age group i.e. 60 years & above (0.563). Similar findings were observed among hospitalized cases (0.540 among 45–60 years and 0.524 among patients aged above 60 years).

As shown in Tables 1 and 2, the utility scores were observed to increase with increase in level of education, ranging from 0.463 to 0.529 among illiterates to 0.630 to 0.724 among graduates and postgraduates. Moreover, HRQoL among outpatients was observed to be lowest among richer income groups (ranging from 0.548 to 0.587), and highest for poorest income groups (0.725). A similar pattern was observed among hospitalized cases.

### Cancer related HRQoL by clinical characteristics

As depicted in Tables 1 and 2, a significant decline in the HRQoL was observed with an increase in the severity of cancer stage (*p* value < 0.001). The poorest HRQoL was observed in stage IV cancer patients, ranging from 0.516 to 0.557, followed by stage III (0.609 to 0.689), stage II (0.677 to 0.713), and stage I (0.638 to 0.748) among inpatients and outpatients, respectively.

The outpatients who had undergone surgery were observed to have significantly poorer quality of life (0.529, *p* value < 0.001) than patients on other treatment modalities like palliative care (0.540), combination therapy (0.604), radiotherapy (0.611), chemotherapy (0.644), diagnostics (0.686), maintenance therapy (0.741) and hormone therapy (0.801). However, indoor cancer patients who were on maintenance therapy (-0.276) and palliative care reported poorer quality of life (0.247) in comparison to patients on other treatment modalities including combination therapy (0.413), diagnostics (0.451), hormone therapy (0.488), surgery (0.518), chemotherapy (0.587), radiotherapy (0.621) and immunotherapy (0.726) etc.

Further, cancer patients with adverse effects had lower quality of life (0.627 & 0.544) in comparison to patients with no adverse effects (0.835 & 0.637) among both outpatients and inpatients respectively. Also, patients who were disease free reported significantly better quality of life (0.691 among outpatients and 0.553 among inpatients) in contrast to patients with progressive disease (0.553 among outpatients and 0.420 among inpatients). Furthermore, patients who were hospitalized and on the first line of treatment reported a higher utility score of 0.572, in contrast to those on subsequent lines of treatment, with scores of 0.417, 0.444, and 0.416 for the second, third, and fourth lines of treatment, respectively.

Mean EQ-5D-5L utility scores showed significant differences across various categories, including education, marital status, treatment, stage of disease, treatment response, and the adverse effects among both outpatients and inpatients. In addition, mean EQ-5D-5L utility scores varied significantly across different age groups, gender, residential status and income quintile among outpatients. Significant differences were observed in EQ-5D-5 L scores based on the type of hospital, duration of hospitalization, and the line of treatment among inpatients.

### Factors associated with HRQoL among outpatients

The utility score was found to decrease by 0.001 (B = -0.001) with every one unit increase in life year of the cancer patient (Table 3). The utility score increased significantly with level of education (B = 0.043 among patients having primary and middle level education versus B = 0.115 among graduates and postgraduates). In comparison to cancer patients in urban settings, individuals from slum areas reported a notably diminished quality of life (B = -0.098). The utility scores were observed to be significantly lower among richer income groups as compared to the poorest (B = -0.081 among rich and B = -0.058 among richest income groups). The patients on hormone therapy (B = 0.076) had better HRQoL as compared to patients on chemotherapy. However, palliative care (B=-0.137) and surgery (B=-0.110) were significantly associated with poorer HRQoL. Further, stage 4 cancer patients were observed to have significantly lower quality of life (B = -0.118) as compared to stage 1 cancer cases. As compared to first line therapy, cancer patients on third line of treatment were found to have significantly poorer quality of life (B = -0.146). Further, patients who were in progressive disease state were found to have significantly poorer quality of life (B = -0.0081) in comparison to disease-free survival patients. Absence of adverse effects was also significantly associated with higher quality of life (B = 0.113) in comparison to presence of adverse effects in cancer patients.

**Table 3:** Factors influencing health-related quality of life among cancer patients seeking outpatient treatment in India

Parameter		B	Std. Error	95% Confidence Interval		***p***-value*
				Lower	Upper	
**(Intercept)**		0.745	0.037	0.673	0.817	0.000
**Age**		-0.001	0.000	-0.002	-0.001	**0.001**
**Gender (Reference- Male)**	Female	0.015	0.011	-0.006	0.036	0.156
**Area of Residence (Reference- Urban)**	Rural	0.006	0.011	-0.017	0.028	0.618
	Slum	-0.098	0.042	-0.181	-0.016	**0.019**
**Education (Reference- No Education)**	Primary & Middle	0.043	0.013	0.018	0.069	**0.001**
	Up to Senior Secondary	0.096	0.014	0.068	0.124	**0.000**
	Graduation & above	0.115	0.020	0.076	0.154	**0.000**
**Marital Status (Reference-Unmarried)**	Married	0.012	0.024	-0.036	0.059	0.624
	Separated/Divorced	-0.072	0.062	-0.193	0.049	0.244
	Widow/Widower	0.028	0.030	-0.031	0.087	0.348
**Wealth Quintile (Reference- Poorest)**	Poor	-0.023	0.015	-0.052	0.006	0.124
	Middle	-0.023	0.016	-0.053	0.008	0.150
	Rich	-0.081	0.015	-0.111	-0.050	**0.000**
	Richest	-0.058	0.016	-0.090	-0.027	**0.000**
**Type of Cancer (Reference-Solid)**	Haematological	0.104	0.082	-0.057	0.266	0.206
**Type of Treatment (Reference- Chemotherapy)**	Radiotherapy	-0.018	0.018	-0.053	0.017	0.313
	Palliative care	-0.137	0.031	-0.197	-0.076	**0.000**
	Surgery	-0.110	0.019	-0.147	-0.072	**0.000**
	Combination Therapy**	-0.001	0.014	-0.028	0.026	0.944
	Maintenance Therapy	0.097	0.154	-0.205	0.399	0.531
	On diagnostic workup	0.051	0.088	-0.121	0.224	0.559
	Hormone Therapy	0.076	0.030	0.016	0.136	**0.013**
	Others (clinical follow up)	0.062	0.024	0.016	0.109	**0.009**
**Line of Treatment (Reference -First Line)**	Second Line	-0.001	0.018	-0.035	0.034	0.977
	Third Line	-0.146	0.050	-0.245	-0.047	**0.004**
	Fourth Line	0.056	0.191	-0.319	0.431	0.770
**Cancer Stage (Reference - Stage I)**	Carcinoma	-0.128	0.188	-0.497	0.241	0.498
	Stage II	-0.011	0.021	-0.053	0.031	0.609
	Stage III	-0.023	0.020	-0.062	0.015	0.231
	Stage IV	-0.118	0.021	-0.159	-0.078	**0.000**
**Response to Treatment (Reference -Disease Free survival)**	Progressive	-0.081	0.027	-0.133	0.028	**0.003**
	Ongoing	0.016	0.015	-0.013	0.046	0.273
**Adverse Effect (Reference - With Adverse Effect)**	Without Adverse Effect	0.113	0.021	0.071	0.155	**0.000**

*Level of significance at p- value less than 0.05, **Combination therapy– Chemotherapy + Radiotherapy, Surgery + Radiotherapy, Surgery + Chemotherapy, Surgery + Chemotherapy + Radiotherapy. Ongoing treatment-response cannot be assessed among patients on active oncology treatment

### Factors associated with HRQoL among hospitalized patients

The utility score was observed to decrease by 0.002 (B = -0.002) with every one unit increase in life year of the cancer patient (Table 4). The patients having higher educational status (graduates & post graduates) reported significantly higher HRQoL (B = 0.075, *p* < 0.001) as compared to illiterate patients. As compared to first line therapy, cancer patients on second line therapy reported significantly lower HRQoL (B =- 0.065, *p* < 0.008). The utility scores were found to be significantly higher among cancer patients without adverse events (B = 0.085, *p* < 0.001). The patients hospitalized in semi-private hospitals had higher HRQoL (B = 0.231, *p* < 0.001), as against those admitted in public hospitals. The patients on surgery (B = -0.127), combination therapy (B = -0.133), diagnostic work up (B = -0.144), palliative care (B = -0.258) and maintenance therapy (B = -0.702) were observed to be significantly associated with poorer quality of life paralleled to chemotherapy. Further, patients who were in progressive disease state were found to experience significantly lower HRQoL (B = -0.149) relative to disease free survival patients.

**Table 4:** Factors influencing health-related quality of life among hospitalized cancer cases in India

Parameter		B	Std. Error	95% Confidence Interval		***p***-value*
				Lower	Upper	
**(Intercept)**		0.500	0.038	0.426	0.574	0.000
**Age**		-0.002	0.000	-0.003	-0.001	**0.000**
**Hospital Days**		-0.003	0.002	-0.006	0.000	**0.060**
**Type of Hospital (Reference - Public)**	Semi-private	0.231	0.016	0.200	0.263	**0.000**
**Education (Refernce- No Education)**	Primary & Middle	0.031	0.020	-0.009	0.071	0.133
	Up to Senior Secondary	0.031	0.021	-0.009	0.072	0.128
	Graduation & above	0.075	0.023	0.030	0.120	**0.001**
**Type of Cancer (Reference-Solid)**	Haematological	-0.033	0.027	-0.086	0.020	0.218
**Type of Treatment (Reference- Chemotherapy)**	Radiotherapy	-0.025	0.033	-0.090	0.039	0.440
	Palliative care	-0.258	0.056	-0.367	-0.149	**0.000**
	Surgery	-0.127	0.033	-0.192	-0.062	**0.000**
	Combination Therapy**	-0.133	0.024	-0.181	-0.085	**0.000**
	Maintenance Therapy	-0.702	0.151	-0.998	-0.406	**0.000**
	On diagnostic workup	-0.144	0.037	-0.216	-0.072	**0.000**
	Hormone Therapy	-0.163	0.184	-0.523	0.198	0.377
	Immunotherapy	0.098	0.062	-0.022	0.219	0.110
	Others (clinical follow up)	-0.136	0.029	-0.193	-0.080	**0.000**
**Line of Treatment (Reference - First Line)**	Second Line	-0.065	0.025	-0.114	-0.017	**0.008**
	Third Line	0.037	0.062	-0.084	0.158	0.550
	Fourth Line	0.117	0.104	-0.086	0.321	0.258
**Response to Treatment (Reference– Disease free survival)**	Progressive	-0.149	0.040	-0.227	-0.071	**0.000**
	Ongoing	0.016	0.024	-0.031	0.064	0.504
**Adverse Effect (Reference - With Adverse Effect)**	Without Adverse Effect	0.085	0.025	0.037	0.133	**0.001**

*Level of significance at p- value less than 0.05, **Combination therapy– Chemotherapy + Radiotherapy, Surgery + Radiotherapy, Surgery + Chemotherapy, Surgery + Chemotherapy + Radiotherapy. Ongoing treatment-response cannot be assessed among patients on active oncology treatment

### Cancer site specific utility scores among patients seeking outpatient and indoor treatment

The stratified analysis was conducted to calculate utility scores based on primary cancer site for patients receiving both hospitalized and outpatient treatment, as presented in Tables 5 and 6. Significant differences (*p* value < 0.001) were noted between utility scores across various cancers. Among outpatients, utility scores ranged from 0.305 (bone cancer) to 0.782 (Leukemia). For hospitalized cases, the lowest utility score was observed for multiple myeloma (0.255), while the highest was for testicular cancer (0.771). Site-specific utility scores, using both EQ-5D-5L and EQ-VAS, stratified by the cancer site for patients who sought outpatient and hospitalized treatment, are detailed in Tables 5 and 6, respectively.

**Table 5:** Primary-site-specific mean health-related quality of life (HRQoL) score for cancer-related outpatient treatment

Primary site of Cancer	Sample size N (%)	Mean HRQoL score		VAS Score	
		Mean (95%CIs)	***p***-value	Mean (95%CIs)	***p***-value
Bladder cancer	74 (0.8%)	0.579(0.498,0.66)	**< 0.001**	64.62(61.02,68.22)	**< 0.001**
Bone cancer	146 (1.5%)	0.305(0.222,0.388)		56.2(53.34,59.05)	
Brain and other nervous system cancers	100 (1.0%)	0.555(0.467,0.643)		62.77(59.58,65.96)	
Breast cancer	2303(23.6%)	0.72(0.709,0.732)		67.7(67.03,68.38)	
Cancer of unknown primary site	44 (0.5%)	0.52(0.376,0.664)		58.41(52.3,64.52)	
Cervical and Uterine cancers	654 (6.7%)	0.613(0.583,0.643)		65.34(63.94,66.74)	
Colorectal cancer	457 (4.7%)	0.616(0.584,0.648)		58.24(56.76,59.72)	
Head and Neck cancer	454 (4.7%)	0.581(0.547,0.615)		62.35(60.87,63.84)	
Oral cancer	658 (6.7%)	0.584(0.555,0.612)		62.72(61.49,63.94)	
Kidney and ureter cancer	65 (0.7%)	0.551(0.453,0.65)		58.45(54.19,62.7)	
Leukemia	1167 (12%)	0.782(0.766,0.798)		70.95(69.86,72.04)	
Lung cancer	743 (7.6%)	0.58(0.55,0.609)		60.95(59.63,62.26)	
Lymphoma	434 (4.4%)	0.651(0.616,0.686)		64.69(62.93,66.44)	
Multiple Myeloma	347 (3.6%)	0.63(0.591,0.668)		65.84(63.92,67.76)	
Ovarian cancer	745 (7.6%)	0.701(0.676,0.726)		65.68(64.21,67.15)	
Pancreatic and Biliary cancers	365 (3.7%)	0.576(0.537,0.614)		54.16(52.74,55.58)	
Prostate cancer	118 (1.2%)	0.624(0.556,0.692)		60.17(57.09,63.25)	
Penile cancer	25 (0.3%)	0.529(0.352,0.706)		64.92(57.81,72.03)	
Skin cancer	36 (0.4%)	0.343(0.153,0.532)		59.31(54.09,64.52)	
Soft tissue tumors	63 (0.6%)	0.564(0.462,0.666)		54.44(49.94,58.95)	
Testicular cancer	81 (0.8%)	0.703(0.618,0.788)		62.89(58.33,67.45)	
Upper GI tract cancers	500 (5.1%)	0.64(0.612,0.668)		57.57(56.26,58.87)	
Other hematological cancers (exc. Lymphomas and Leukemia)	151 (1.5%)	0.633(0.577,0.689)		61.95(58.98,64.93)	
Other cancers	32 (0.3%)	0.804(0.731,0.877)		67.19(60.08,74.29)	

*Cancer categories are based on top 30 cancers as per Global disease burden 2016 estimates for India, Level of significance at p- value less than 0.05

**Table 6:** Primary-site-specific mean health-related quality of life (HRQoL) score among hospitalized cancer patients

Primary site of Cancer	Sample size N (%)	Mean HRQoL score		VAS Score	
		Mean (95%CIs)	***p***-value	Mean (95%CIs)	***p***-value
Bladder cancer	19 (0.8%)	0.579(0.371,0.788)	**< 0.001**	56.84(49.51,64.17)	**< 0.001**
Bone cancer	73 (3.2%)	0.392(0.271,0.513)		54(50.6,57.4)	
Brain and other nervous system cancers	24 (1%)	0.326(0.127,0.525)		47.5(39.29,55.71)	
Breast cancer	309 (13.1%)	0.648(0.614,0.682)		56.8(55.07,58.52)	
Cancer of unknown primary site	17 (0.7%)	0.624(0.499,0.749)		57.06(50.82,63.29)	
Cervical and Uterine cancers	130 (5.5%)	0.55(0.486,0.615)		58.12(55.66,60.57)	
Colorectal cancer	264 (11.2%)	0.595(0.554,0.636)		56.41(54.75,58.07)	
Head and Neck cancer (excluding Oral cavity)	112 (4.7%)	0.533(0.461,0.605)		57.81(55.01,60.62)	
Oral cancer	201 (8.5%)	0.507(0.462,0.552)		58.76(56.72,60.79)	
Kidney and ureter cancer	18 (0.8%)	0.637(0.493,0.782)		57.22(49.44,65)	
Leukemia	196 (8.3%)	0.562(0.508,0.616)		53.65(51.63,55.66)	
Lung cancer	144 (6.1%)	0.488(0.416,0.56)		54.55(52.09,57.01)	
Lymphoma	166 (7%)	0.562(0.494,0.63)		58.1(55.62,60.59)	
Multiple Myeloma	71 (3%)	0.255(0.145,0.365)		49.3(44.77,53.82)	
Ovarian cancer	163 (6.9%)	0.586(0.531,0.641)		54.17(51.65,56.69)	
Pancreatic and Biliary cancers	92 (3.9%)	0.499(0.425,0.572)		51.58(48.58,54.58)	
Prostate cancer	21 (0.9%)	0.54(0.35,0.729)		54.29(48.98,59.59)	
Penile cancer	9 (0.4%)	0.327(-0.091,0.745)		57.78(45.91,69.64)	
Skin cancer	10 (0.4%)	0.289(-0.06,0.639)		56(47.11,64.89)	
Soft tissue tumors	28 (1.2%)	0.532(0.364,0.7)		54.29(48.94,59.63)	
Testicular cancer	59 (2.5%)	0.771(0.702,0.84)		57.97(53.19,62.75)	
Upper GI tract cancers	203 (8.6%)	0.579(0.527,0.631)		53.23(51.25,55.21)	
Other hematological cancers (exc. Lymphomas and Leukemia)	12 (0.5%)	0.229(-0.083,0.541)		40.83(31.66,50)	
Other cancers	16 (0.7%)	0.601(0.414,0.787)		53.13(46.83,59.42)	

*Cancer categories are based on top 30 cancers as per Global disease burden 2016 estimates for India, Level of significance at p- value less than 0.05

## Discussion

Despite the advent of advanced therapeutic technologies which have significantly improved survival, achieving cancer-free status does not directly imply an improved quality of life [47]. The frequently considered efficacy criteria of cancer therapy often prove insufficient, lacking a comprehensive approach to the entire disease process, treatment, and overall well-being. As a result, it is imperative to account for the changes in HRQoL with the newer therapies, apart from their impact on survival [48, 49].

It is imperative to shift the objective of cancer treatment from solely achieving successful health outcomes to a more encompassing goal of enhancing the overall HRQoL for patients. A deeper comprehension of HRQoL in cancer patients is instrumental in enriching the lived experience of cancer survivors, emphasizing the importance of adding more life to the added years, rather than merely extending the years of life. Limited studies are available across the globe as well as from India that comprehensively assess HRQoL among cancer patients. None of the studies conducted so far have reported quality of life scores according to typer of cancer site, stage, treatment and response. Majority quality of life assessment studies conducted in India have focussed on single type of cancer with small sample sizes, thus cannot be generalizable to all cancer patients in India [50, 51, 52].

The findings of our study suggest that the HRQoL of cancer patients declines as their income level increases. A previous study conducted in India also revealed an escalating gradient in the self-reported morbidity with higher socioeconomic status [51, 53]. This suggests that individuals with higher income tend to report a diminished quality of life for a similar health condition—a phenomenon termed as positional objectivity [33]. It is plausible that wealthier individuals may be more health-conscious, leading them to assess their quality of life in relation to their health differently than those with lower incomes. Conversely, individuals with lower economic status may face unmet basic life needs, placing “health” lower in their priority ranking. Consequently, the wealthier individuals perceive themselves to have a poorer quality of life compared to their less affluent counterparts. Further, poorer HRQoL was observed among elderly patients among both outpatients and inpatients which indicate that these frail patients have poor tolerance to cancer treatment, slow recovery from adverse effects and hence attempts should be made for de-escalation of treatment in this elderly group to improve the HRQoL. The HRQoL was also found to be better among graduates and postgraduates (0.724 and 0.630 among outpatients and inpatients respectively). Higher education is associated with better awareness about the disease, treatment options and outcomes. The ability to make informed decisions improves the HRQoL in educated groups. Contrary to this ignorance breeds more fears and inability to make decisions. Thus, it is important to generate awareness about disease, treatment and outcomes in the illiterate cancer patients through social workers or Non-governmental organizations to improve their HRQoL.

Given the large sample size, our study is powered to provide valid estimates of HRQoL for 12 specific cancers in India, with a 5% margin of error; and top 20 cancers with a 10% margin of error and a 95% confidence interval.

### Policy implications

Quality-adjusted life year (QALYs) has been recommended as a metric for valuation of health outcomes for economic evaluations in India [54]. In order to compute QALYs, the valuation of HRQoL for various health states is necessary. However, collecting primary data for estimating utility scores is a time-consuming and resource-intensive process. The HRQoL database developed in this study would be immensely beneficial for expediting HTA analyses. This is particularly crucial as HTA studies specifically require information on utility scores. Therefore, the current research makes a substantial contribution to the current body of evidence by providing separate utility scores specific to cancer site and cancer stage.

The clinical trials should not only assess the safety and survival end points but also incorporate the indicator of quality of life so as to choose best intervention for cancer care. Further, interventions causing minimal adverse effects should be explored as majority patients having adverse effects reported poorer quality of life. As financial constraints are major problem for the patients, financial toxicity and the impact on quality of life should be taken into account before recommending cost-intensive treatments to poor patients in palliative settings.

### Strengths

We would like to highlight some of the methodological strengths of our study. Firstly, our patient cohort was sourced from seven healthcare facilities, encompassing the largest volume of oncology patients nationwide. Given that the HRQoL assessment is influenced by factors such as culture, ethnicity, region, and socio-demographic characteristics, the selected study hospitals provide a broad geographical representation. Therefore, the patient population, selected through systematic random sampling, is indicative of the diversity within the Indian population. Secondly, utility scores were computed using country-specific tariff values i.e. the Indian value-set [45]. Thirdly, it is the first study to ascertain HRQoL and its predictors in a substantially large sample of 12,148 cancer patients. Fourth, our study provides valuable information to identify determinants of HRQoL.

### Limitations

We acknowledge certain limitations in our study. Firstly, as present study was a cross-sectional survey, therefore we could not assess the HRQoL of patients pre and post intervention. However, since the sample of patients was heterogeneous in nature, the data pertaining to HRQoL represented all types of cancer patients on different treatment modalities. Secondly, we have assessed the HRQoL among hospitalized cases at the time of recruitment and did not record the quality of life on each of day of hospitalization till discharge.

## Conclusion

In conclusion, this study provides empirical evidence on HRQoL among cancer patients, as well as significant predictors of the HRQoL. Older age, lower educational status, chemotherapy, palliative care and surgery, stage of cancer, progressive disease were associated with poor HRQoL. The results of the present study should be used in devising individualized treatment plans, enhancing patient care, improving compliance and follow-up.

## Data Availability

The data underlying this article are available in the article and in its online supplementary material. The datasets and analysis will be available upon request. The study investigators retain ownership of their data. Any requests for access to data should be made directly to study investigator.
